# EnRoot: a narrow-diameter, inexpensive and partially 3D-printable minirhizotron for imaging fine root production

**DOI:** 10.1186/s13007-019-0489-6

**Published:** 2019-08-28

**Authors:** Marie Arnaud, Andy J. Baird, Paul J. Morris, Angela Harris, Jonny J. Huck

**Affiliations:** 1 School of Geography, University of Leeds, Leeds, LS2 9JT UK; 2 School of Environment and Development, University of Manchester, Manchester, M13 9PL UK

**Keywords:** Minirhizotron, Root production, Belowground carbon, Fine roots, 3D minirhizotron, Root monitoring, Belowground biomass, Carbon sequestration, Root dynamics

## Abstract

**Background:**

Fine root production is one of the least well understood components of the carbon cycle in terrestrial ecosystems. Minirhizotrons allow accurate and non-destructive sampling of fine root production. Small and large scale studies across a range of ecosystems are needed to have baseline data on fine root production and further assess the impact of global change upon it; however, the expense and the low adaptability of minirhizotrons prevent such data collection, in worldwide distributed sampling schemes, in low-income countries and in some ecosystems (e.g. tropical forested wetlands).

**Results:**

We present EnRoot, a narrow minirhizotron of 25 mm diameter, that is partially 3D printable. EnRoot is inexpensive (€150), easy to construct (no prior knowledge required) and adapted to a range of ecosystems including tropical forested wetlands (e.g. mangroves, peatlands). We tested EnRoot’s accuracy and precision for measuring fine root length and diameter, and it yielded Lin’s concordance correlation coefficient values of 0.95 for root diameter and 0.92 for length. As a proof of concept, we tested EnRoot in a mesocosm study, and in the field in a tropical mangrove. EnRoot proved its capacity to capture the development of roots of a legume (*Medicago sativa*) and a mangrove species (seedlings of *Rhizophora mangle*) in laboratory mesocosms. EnRoot’s field installation was possible in the root-dense tropical mangrove because its narrow diameter allowed it to be installed between larger roots and because it is fully waterproof. EnRoot compares favourably with commercial minirhizotrons, and can image roots as small as 56 µm.

**Conclusion:**

EnRoot removes barriers to the extensive use of minirhizotrons by being low-cost, easy to construct and adapted to a wide range of ecosystem. It opens the doors to worldwide distributed minirhizotron studies across an extended range of ecosystems with the potential to fill knowledge gaps surrounding fine root production.

## Background

Root production is one of the least studied components of terrestrial ecosystems, despite being likely to represent a third of net primary production [6]. Several techniques exist to measure in situ fine root production, but quantifying such subterranean processes remains difficult and often expensive. Minirhizotrons involve the installation of a transparent tube into the soil, into which a camera is inserted periodically to record root development [5]. Net root production is estimated by calculating changes in root diameters and lengths between successive images [7].

Minirhizotrons have proven to be accurate for root production measurements [7] and overcome limitations associated with other methods because: (i) the same roots and soil profile are sampled repeatedly, reducing the spatial component of experimental error [4]; (ii) root production and mortality are measured simultaneously, minimising the likelihood of missing any roots with a fast turnover (appearance, growth and death, [3]); (iii) they do not use artificial soil substrate, which might modify the root production unlike ingrowth cores [13]; and (iv) they are non-destructive; once the minirhizotron tubes have been installed, no subsequent disturbance is required to take repeated measurements [9]. The minimal disturbance is advantageous for both mesocosm studies and long-term field experiments.

Minirhizotrons do, however, have limitations. They are expensive [12] and lack a standardised design; consequently, they are not used in worldwide distributed sampling schemes, such as RAINFOR and GEM networks plots [10] and are rarely used in low-income countries. Additionally, they are not well adapted to wetland conditions [5], including tropical forested wetlands. While working in waterlogged wetland soils or during heavy rain (e.g. monsoon), commercial minirhizotron cameras, being non-waterproof, can be easily damaged. Large above- and below-ground roots, also make the installation of commercially-available minirhizotron tubes (with diameters of over 50 mm) in tropical forested wetlands difficult or impossible. As a result, minirhizotrons are either not used (e.g., in mangroves) or are located away from tree trunks, resulting in potentially unrepresentative fine root production estimates. Narrow minirhizotrons would be easier to install. Hand-made minirhizotrons might partially overcome the price limitation, but are usually produced in small numbers for a specific application, are hard to construct (prior knowledge is required), and often are not well documented. There is a need for a minirhizotron that overcomes the limitations identified above and that can be made cheaply and easily to a reproducible specification that allows comparison among and between sites and ecosystems.

Here, we report the development and testing of a narrow, waterproof and inexpensive minirhizotron that can be repeatedly and easily made to a standardised specification: EnRoot. With its narrow diameter and waterproof camera, EnRoot is easy to install and suitable for a large range of ecosystems, including tropical forested wetlands. The material costs—including the camera—are less than €150 per unit. EnRoot is easy to assemble and to reproduce to a standard with its 3D printable components costing less than €35, and has similar imaging capabilities to commercial minirhizotrons.

## Results and discussion

### The new minirhizotron system: EnRoot

#### Description and set up of EnRoot

**Fig. 1:**
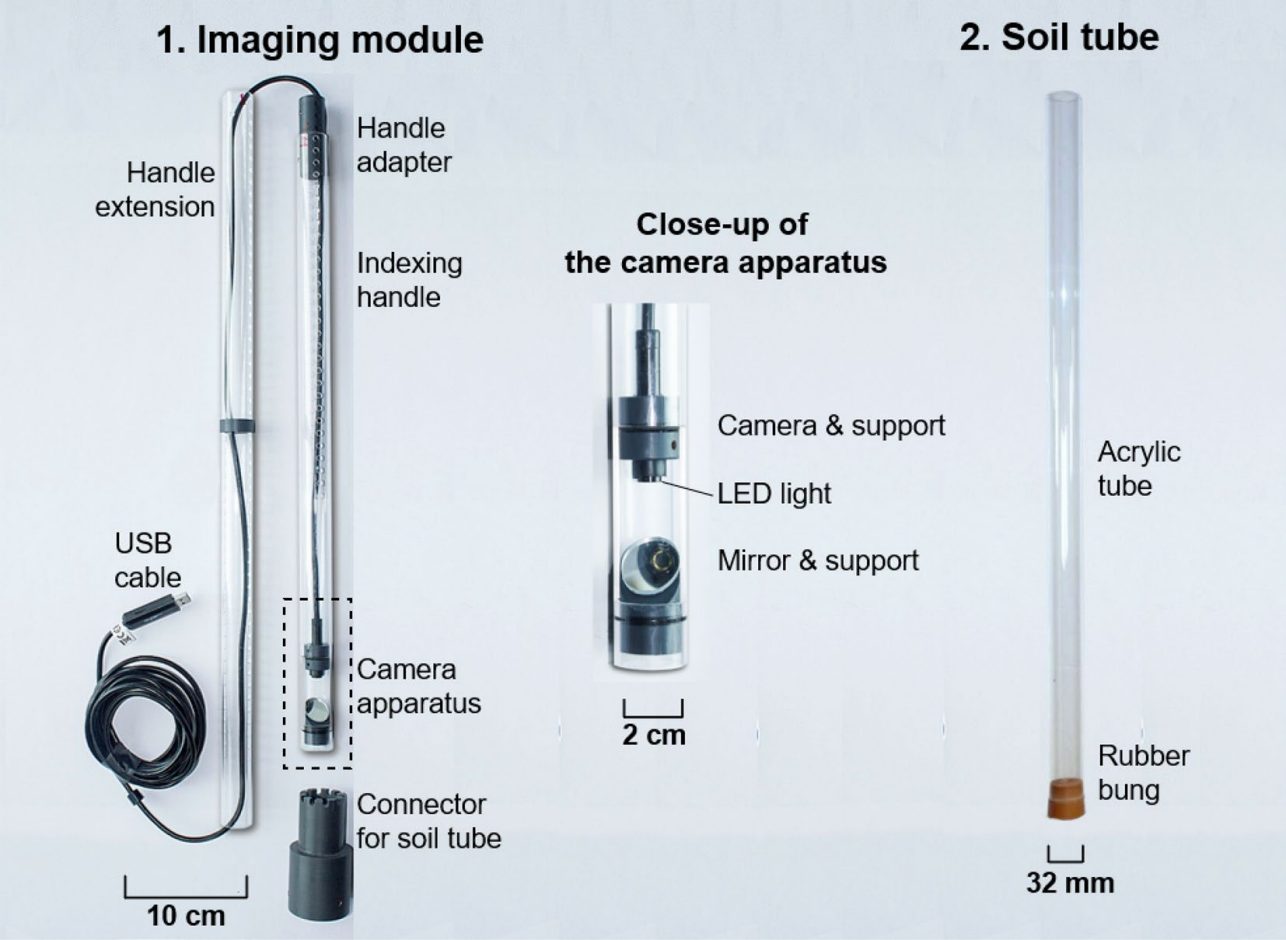
EnRoot’s components. All the grey plastic components are 3D printable

**Fig. 2:**
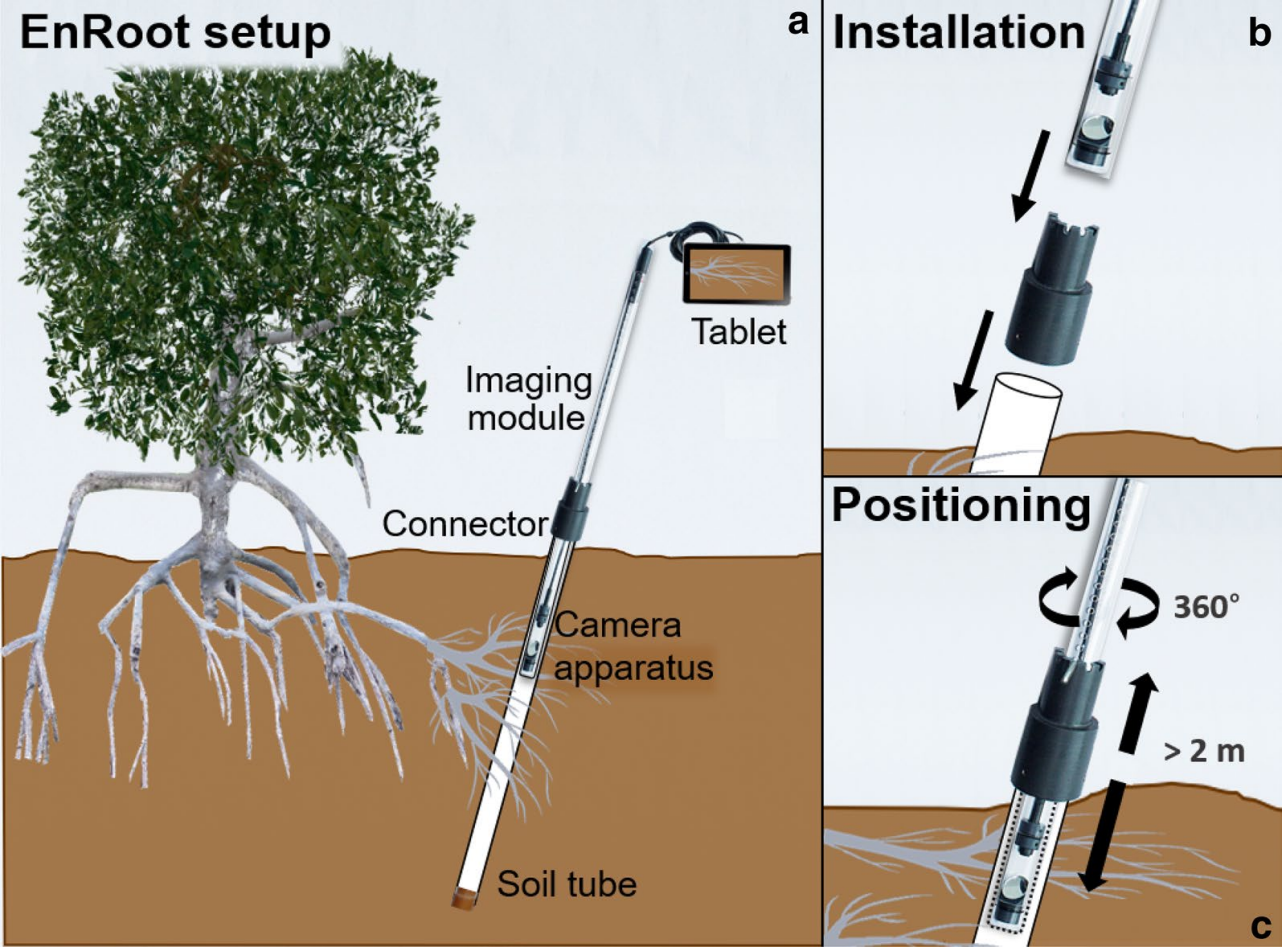
EnRoot’s setup and usage

**Fig. 3:**
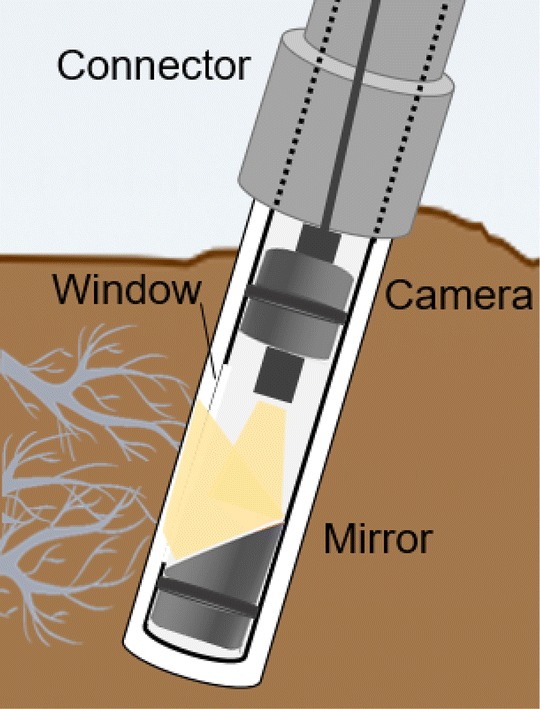
The design of the camera apparatus used in EnRoot

EnRoot has two main components—an imaging module and a soil tube (Figs. 1, 2). Both are very narrow; the module has an outside diameter of 25 mm, and the soil tube an outside diameter of 32 mm. EnRoot’s soil tube is left permanently buried in the soil to allow the development of roots around it (Fig. 2a). The tube is made from clear acrylic (2 mm thick; purchased from http://theplasticshop.co.uk) and has a rubber bung fitted in its base (Fig. 1). The imaging module is composed of an indexing handle and a camera apparatus (Fig. 1); both have and inside diameter of 21 mm, and an outside diameter of 25 mm. The camera apparatus is 15 cm long and the indexing handle 45 cm long. The handle extension length is adjustable so that the camera apparatus can reach the bottom of soil tubes. The holes of the indexing handle and its extension are drilled every centimetre with a pillar drill (AJVBM 4, Ajax) using a drill bit of 4 mm. The distance between the drilled holes can be adjusted to take pictures with no or a range of overlaps. The camera apparatus has a window through which the roots are directly observed with a camera (Potensic^®^ 2-in-1 USB Endoscope with LEDs) and a mirror orientated at 40° relative to the soil tube’s long axis (Fig. 3).

**Fig. 4:**
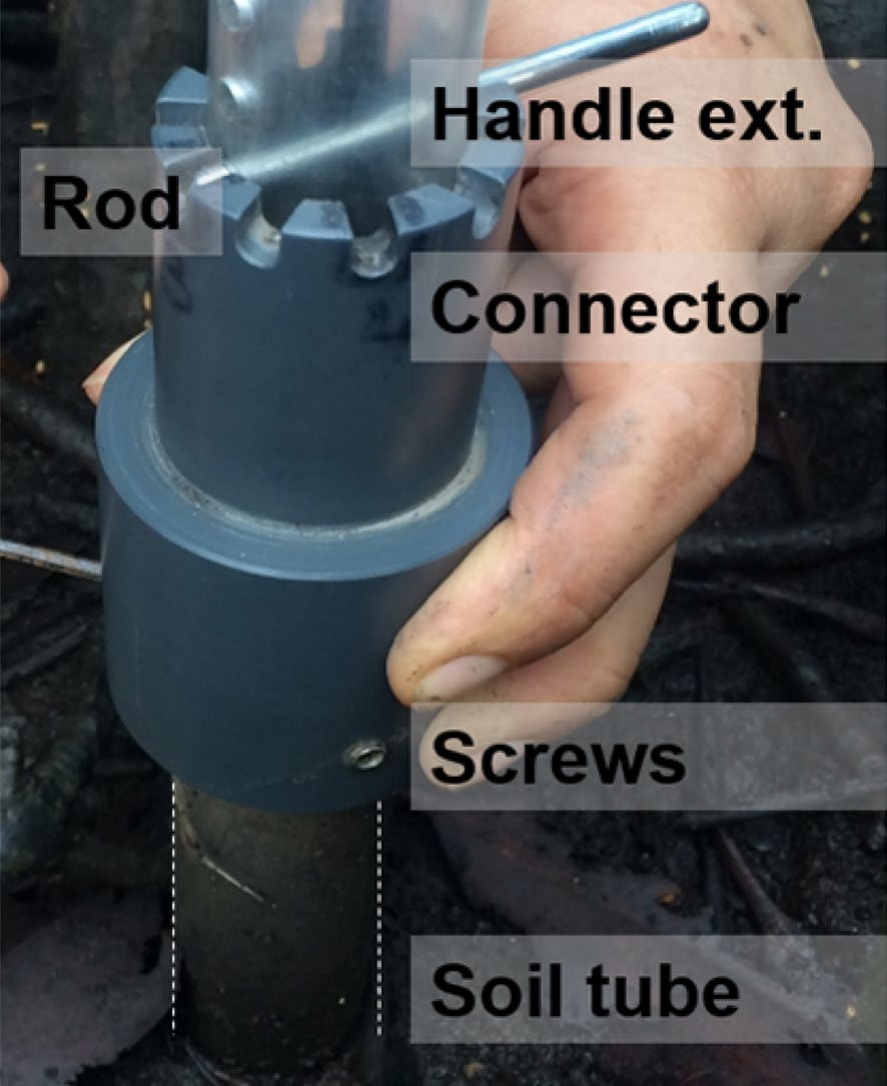
EnRoot’s indexing handle and soil tube connector allow images to be taken at precise depths and radial directions within the soil tube. The screws hold the connector in place

The imaging module is inserted into the soil tube, and the full circumference and length of the tube (around where the roots are developing) is imaged by incrementally rotating and moving up and down the imaging module within the soil tube (Figs. 2c, 4). The position of the imaging system can be recorded by inserting a metal rod at a known position into (i) the connector castellated every 5 mm for a 360° coverage, and (ii) the indexing handle perforated every centimetre to reach any depth in the soil tube (Fig. 4). The record of the position allows repeated measurements of the same roots and soil area over time.

**Fig. 5:**
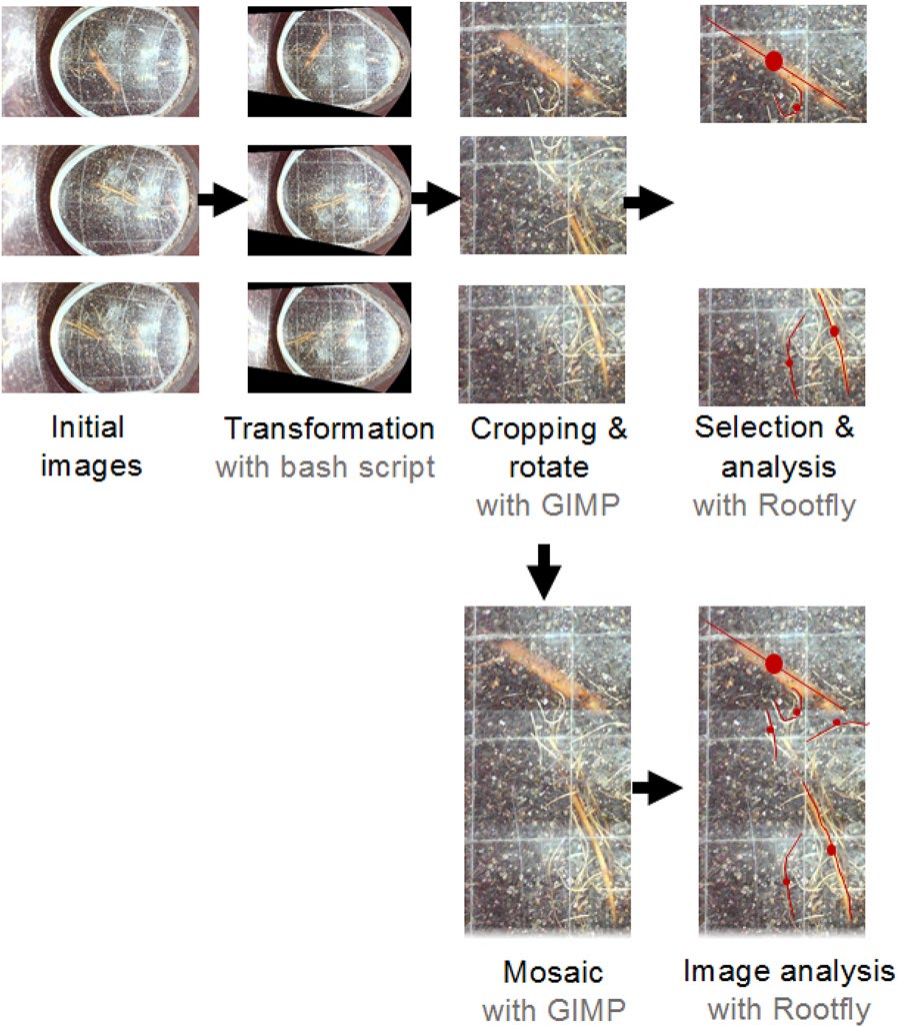
EnRoot’s image processing and analysis. The initial images are transformed with EnRoot’s bash script, then cropped and rotated in batch with GIMP. A selection of those images can be analysed with Rootfly or if the images of the full tube are required, the images can be assembled as a mosaic (with GIMP or other software) and analysed with Rootfly

Once the minirhizotron is set up, EnRoot is connected to a computer, a tablet or a smartphone via its in-built USB cable (Fig. 2). No extra source of power is required. Roots are observed in real-time on the monitor’s screen and saved with an image-acquisition program. We used the Smart Camera software, which is the software provided with the endoscope camera, but any image-acquisition software can operate the camera (e.g. digiCamControl, simpleCV or VideoCapture). The collected images are corrected using a geometric transformation to compensate for distortion from the cylindrical soil tube and the camera lens; correction is automated using the EnRoot bash script that we developed and have made freely available (Additional file 1, Fig. 5). EnRoot’s bash script uses GDAL [2] and a Python script (included in the repository). A step by step guide to install GDAL and a guide to use the bash script is provided in Additional file 1. The images are then cropped. We recommend using the bash mode of GIMP (http://www.gimp.org) to crop the images (< 1 s per image). Generally, only every other image is analysed to reduce analysis time (Fig. 5, [7]). Subsampling images from different depths of a minirhizotron tube showed to have little effect on the experimental results provided the numbers of minirhizotron tubes used are sufficient [5, 7]. If the subsampling method is used, the selected images can be readily analysed using any root-analysis software (e.g. Rootfly, WinRHIZO, rhizoTrak or SmartRoot). However, if the user requires a mosaic of images covering the full soil tube, we recommend using software to create panoramas, such as GIMP, Image Composite Editor (Microsoft) or PowerPoint (Microsoft).”

#### Testing EnRoot’s performance

The resolution of EnRoot exceeds requirements for imaging tree roots and is adequate for small roots, such as grass roots. Its maximum resolution is 1600 × 1200 pixels, equivalent to 28 µm per pixel in our setup. Since two pixels are required to identify a root, EnRoot can theoretically detect roots with a minimum size of 56 µm. The camera can also be set to a lower resolution to save disk space, for example at 1280 × 720 pixels, allowing for roots of a minimum size of 74 µm to be imaged.

We developed EnRoot with the aim that anyone can reproduce it easily. The assembly of EnRoot does not require prior training and takes less than an hour. Its components—the connectors, adaptor and two camera-apparatus supports—are 3D printable. The 3D files required to fabricate these are freely available for use and modification (Additional file 2). We printed these components in polylactic acid thermoplastic using the 3D Hubs printing platform (https://www.3dhubs.com). We chose this platform because of its low price and because it is available in 140 countries, making EnRoot reproducible almost everywhere.

### EnRoot’s accuracy and precision

**Fig. 6:**
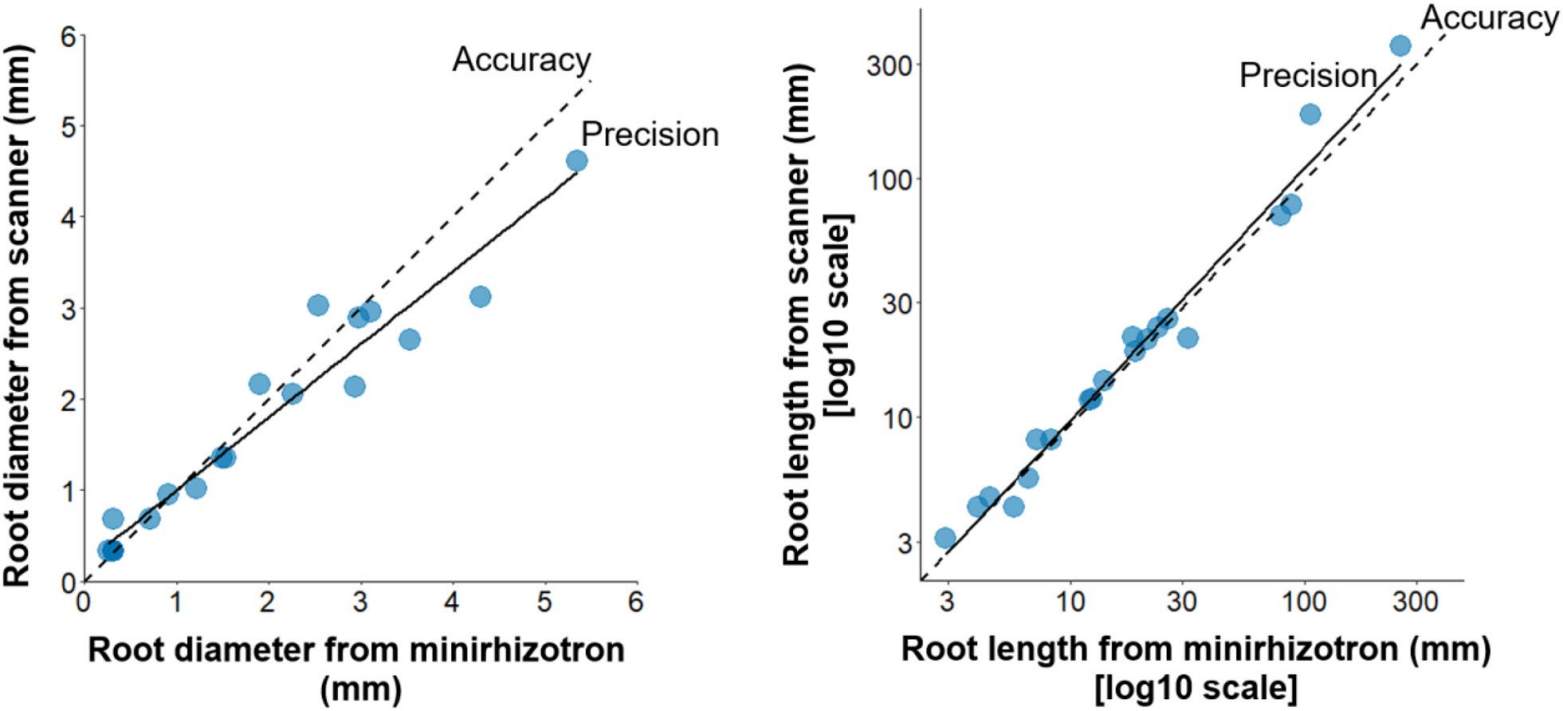
Estimation of root diameter and length with images from EnRoot and a high-resolution scanner. The solid line represents the precision, and the dashed line the accuracy

The measurements of fine root production using minirhizotrons are made by extracting the diameter and length of roots from a series of root images. In order to test EnRoot’s accuracy and precision, we compared root lengths and diameters obtained with EnRoot with measurements from a high-resolution flat scanner (see “Methods”). The root diameters and lengths obtained using EnRoot and the high-resolution scanner were very similar, producing concordance correlation coefficients of 0.95 for root diameter and 0.92 for root length (Lin’s Concordance Correlation Coefficient, Fig. 6). Depending on the descriptive scale used, these values of concordance can be described as moderate to excellent [1, 11]. Despite these encouraging results, there are differences in measurements between the methods (Fig. 6), which we suspect are mainly due to (i) the semi-manual method used by Rootfly to trace and extract root lengths and diameters, and (ii) some alteration of the roots during their attachment to the tube and their installation in the test pot (see “Methods”).

### Using EnRoot in mesocosms and in situ

**Fig. 7:**
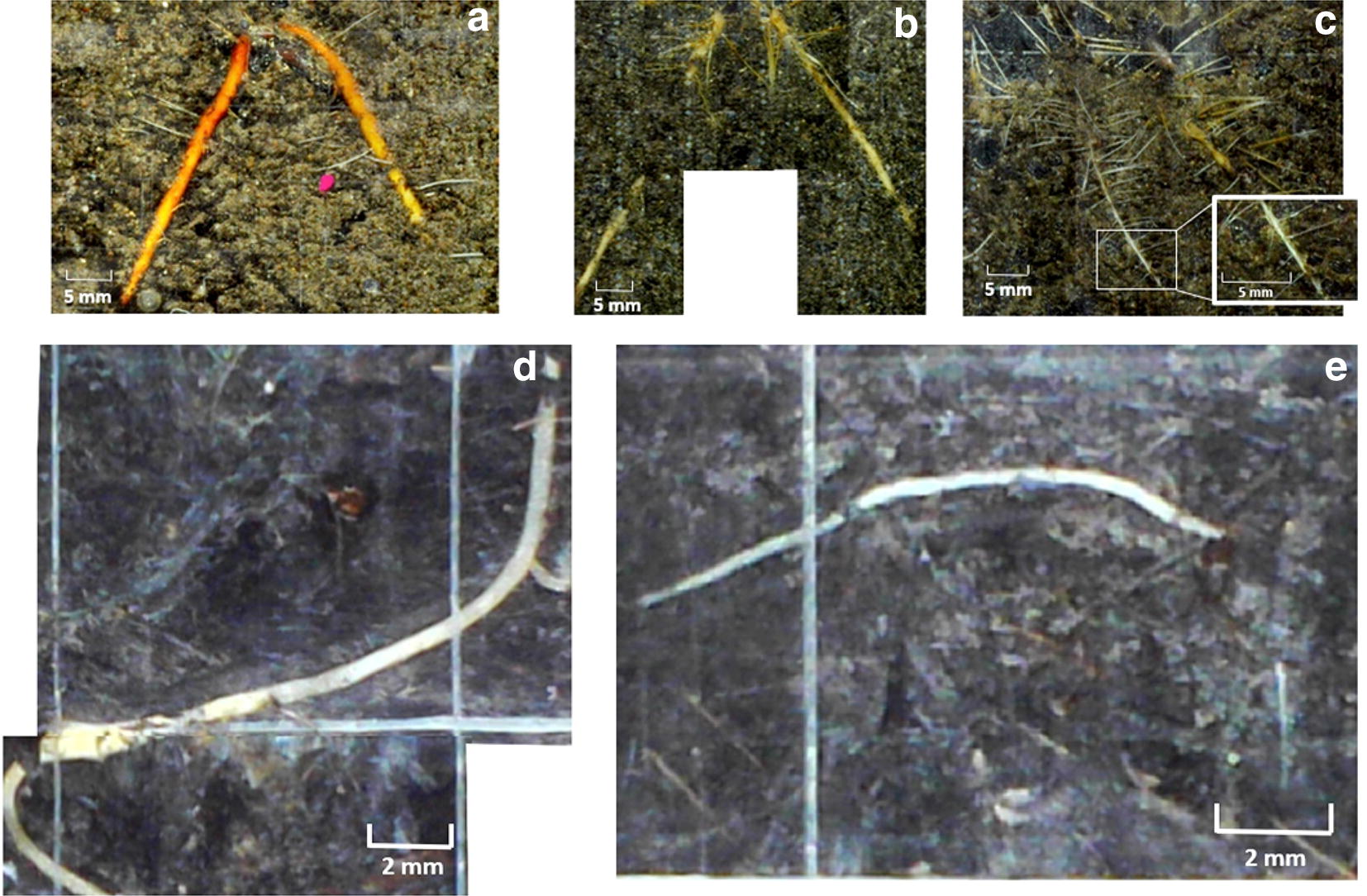
Images of Rhizophora mangle roots with a magnified root in the white box (**a**–**c**) and Medicago sativa roots (**d**, **e**) using EnRoot

We used a series of mesocosm experiments, and installed EnRoot soil tubes in mangroves (see “Methods”), to test the system’s practicality and capacity to image complex rooting systems under different environmental conditions. From the images captured with EnRoot we determined the lengths, diameters, total area and total biomass of the roots in each mesocosm (Table 1). The high resolution, low glare and full colour of the images made it easy to distinguish roots from the substrate (Fig. 7) and delineate root length and diameter using Rootfly (Fig. 7). Specular reflection of the light from the LEDs against the soil tube caused some glare but did not impede the detection and measurement of roots. The initial distortion of the pictures was properly corrected with EnRoot’s bash script.

**Table 1:** Maximum root length and diameter recorded within each mesocosm with the accumulated area of roots imaged with EnRoot and the associated estimated biomass

	Mesocosm									
	1	2	3	4	5	6	7	8	9	10
*Rhizophora mangle*										
Maximum root length (mm)	20.6	35.5	20.3	39	23.8	32.3	36.4	23.5	24.6	42.8
Maximum root diameter (mm)	1.3	1.9	1	2.4	1.3	3	2.0	2.3	2.0	2.2
Total area (mm^2^)	70	77.19	75.6	93.11	102.5	122.62	154.52	190.07	194	244.5
Biomass (g wet weight)	0.10	0.11	0.11	0.13	0.14	0.17	0.22	0.27	0.27	0.34
*Medicago sativa*										
Maximum root length (mm)	40.22	42.65	41.34	36.01	86.33	95.39				
Maximum root diameter (mm)	1.20	0.76	1.17	1.23	1.40	0.99				
Total area (mm^2^)	48.46	50.50	59.83	100.59	219.17	227.38

EnRoot was practical and easy to use. The movement of the imaging module through the soil tube was easily controllable with the indexing handle. It was possible to stop the minirhizotron movement with the help of the connector at any time to capture high resolution images (Fig. 7). The soil tubes remained sealed with no water ingress, and the images were acquired almost instantaneously.

**Fig. 8:**
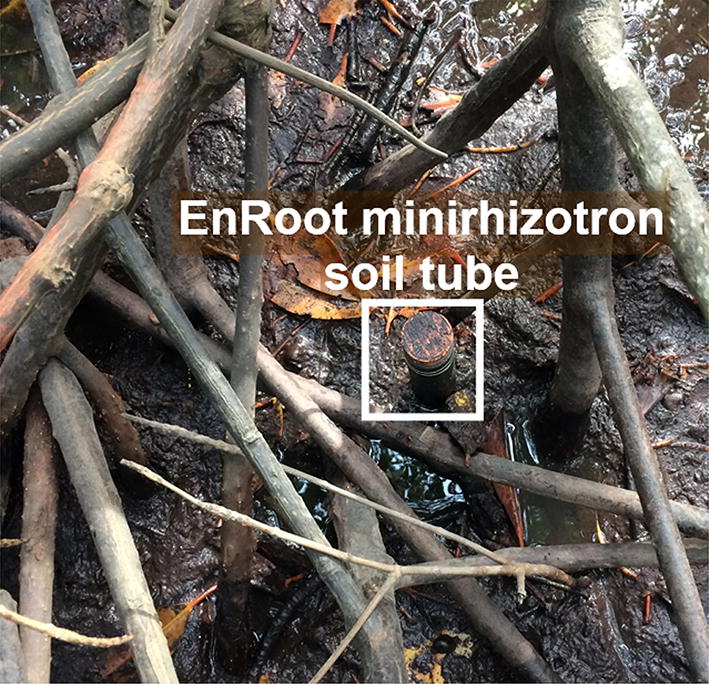
An EnRoot soil tube installed between stilt roots of mangrove trees in Vietnam

In the field, EnRoot soil tubes could easily be installed in-between the aerial and belowground roots of mangroves (a tropical forested wetland, Fig. 8). After 4 months of installation, the roots had developed around the minirhizotron soil tube and were clearly visible in the video we recorded (Additional file 3). There was no water ingress in 59 EnRoot soil tubes after 10 months of installation. Only one tube, that was unknowingly damaged prior to installation, had water ingress. The EnRoot imaging module was accidentally inserted while water was within this soil tube, but it did not cause any damage because the camera is waterproof. The tops of the soil tubes were closed in the field sites with a rubber bung sealed with aquarium sealant. This prevented water ingress from tidal water.

### Comparison of EnRoot specifications with other systems

EnRoot has similar or better specifications than commercial minirhizotrons (see Table 2). The resolution of the images is comparable to other minirhizotrons, but EnRoot is much cheaper than commercial minirhizotrons, at approximately one hundredth to one sixtieth of their price. EnRoot is not, however, suitable for studying hyphae and mycorrhizae. Use of a higher-resolution camera has the potential to extend the system’s capacity to studying such smaller features, albeit at an increased cost. The capture times and the size of the images captured with EnRoot was similar to or better than commercial minirhizotrons. The advantages of EnRoot over commercial minirhizotrons are its: low weight, waterproof camera, small diameter and that it does not require an additional energy source because the system is powered by the computer, tablet or smartphone that it is connected to. EnRoot is also more flexible than commercial minirhizotrons, because it can be easily and freely adapted to different soil tube sizes and image-acquisition software (e.g. digiCamControl, simpleCV or VideoCapture).

**Table 2:** Comparison of EnRoot with the commercial minirhizotrons most cited in the literature

Characteristics	Minirhizotron system			
	EnRoot (this article)	CID bioscience CI-600	Bartz technology BTC-100X	RhizoSystems™, LLC Manual minirhizotron
Price (~ €)	150	14,500	17,500	> 13,500
Waterproof camera	Yes	No	No	No
Theoretical resolution (µm/pixels)^a^	28	42	25	13
Image size (mm)	17 × 12	216 × 196	13.5 × 18	8.4 × 6.3
Capture time (s)	0 to 3	30 to 480^b^	Not indicated	Not indicated
Weight (imaging system only) (g)	250	750	450	6800
Size (mm)	Diameter: > 25 Length: all possible	Diameter: 63.5 Length: 1830	Diameter: 51 Length: 1820	Diameter: 50 Length: 2000
Battery life (h)	No battery needed	> 4	8	11
Magnifier	No	No	Yes	Yes

^a^Theoretical resolution was calculated by dividing the size of the picture by the maximum resolution

^b^For scanning an image of 21.6 × 19.6 cm

## Conclusion

EnRoot opens the minirhizotron method to (i) new usage, particularly in large scale, distributed sampling schemes; (ii) new users, such as researchers in low income countries or those with limited equipment budgets; and (iii) new, carbon-rich ecosystems, such as tropical forested wetlands. The small diameter and waterproofness of EnRoot increases the range of application of minirhizotrons without compromising the quality of the image; EnRoot’s resolution allows theoretical identification of roots with diameters of 56 µm and greater. EnRoot’s lightweight, small diameter tube and no need for external battery offer extra advantages in remote sites. The components of EnRoot are also highly customisable and replacements can be easily built or bought, or 3D printed at low cost.

EnRoot could potentially be enhanced if operated with an external computer program, such as OpenCV-Python, or with rhizoTrak (e.g., for image cropping, creating a mosaic of images and image analysis). Recent progress in the automatic detection and measurement of objects with computer programs means that it is likely that, in the future, the root images could be analysed automatically in order to extract root length, diameter and area directly in the field. Some programs have already been developed in this direction and could be used with EnRoot (e.g., SegRoot or the multiple instance learning algorithms). Such an improvement would save processing image time in the laboratory and remove the lag between image collection and the obtaining of root production data.

Because EnRoot is cheap to build, freely reproducible and easy to use, it has the potential to close our knowledge gap regarding fine root production. Finally, we have focused primarily on root production measurements, but EnRoot could also be used for other applications, such as root phenology studies.

## Methods

### Evaluation of EnRoot for accuracy and precision

To test EnRoot’s accuracy and precision, we used a high-resolution flat scanner (2400 × 4800 dpi, Expression 11000XL, Epson) to scan 20 roots of Red mangrove (*Rhizophora mangle*) with a range of diameters and lengths. The same roots were then wrapped with a transparent plastic film around an EnRoot soil tube (32 mm diameter, 50 cm long) subsequently placed in a test pot (60 cm long and 110 mm diameter, for a total volume of 5702 cm^3^) filled with a peaty soil and then saturated with water. EnRoot was then used to image the same 20 roots. The length and the diameter of the roots were extracted using the freely available software Rootfly [14]. We used Lin’s Concordance Correlation Coefficient to compare the output from both instruments [8]. This metric incorporates both accuracy and precision to quantify the level of agreement between paired measurements and is commonly used to assess bias between instruments or human operators. Accuracy is incorporated through a bias correction factor that represents the gradient of the best-fit line compared to the 1:1 line; while precision is incorporated through the use of Pearson’s Correlation Coefficient (Fig. 6). The value of Lin’s Concordance Coefficient increases towards one as the compared data approach perfect agreement.

### EnRoot trial

The mesocosm experiment was undertaken for 6 months to generate a range of root lengths and diameters representing different stages of fine root production. In the first batch of mesocosms, we mimicked field conditions of mangrove forests. In a greenhouse, 10 *Rhizophora mangle* propagules were planted in ten mesocosm pots (60 cm long and 110 mm diameter, for a total volume of 5702 cm^3^) filled with a mix of sandy and peaty substrate, which was periodically saturated with water. The temperature was maintained at 26 °C with a relative air humidity of 70%. In each mesocosm, an EnRoot soil tube (32 mm diameter and 50 cm long) was installed. After the saplings exhibited first leaf out, we used EnRoot to image the roots of one mesocosm per day at 10 random dates over 6 months. We imaged only the area with roots. We repeated the same experiment with an Alfalfa crop (*Medicago sativa*) in six mesocosms of a peat-only substrate, with temperature maintained between 24 and 26 °C and an average relative air humidity of 30%. Each set of root images was corrected for distortion using EnRoot’s bash script. The images were then cropped using GIMP (http://www.gimp.org) and assembled as a mosaic with GIMP and PowerPoint (Microsoft). The length and diameter of roots within each mesocosm were extracted from mosaics of images using Rootfly. In each mesocosm we identified the longest root and the thickest root, as well as the cumulative area of all the roots imaged. The longest root was defined by the longest continuous segment of root visible. We estimated the fine root biomass of each mangrove mesocosm. Root wet biomass was calculated with a simplified area:biomass coefficient that we calculated for the *Rhizophora mangle* roots. The installation of EnRoot soil tubes was tested in three mangrove sites in the Can Gio Biosphere Reserve in the Mekong Delta in Vietnam where we installed 60 EnRoot soil tubes at 1 m depth. We generated 60 random locations and installed at each an EnRoot soil tube (1.2 m long, so 0.2 m left above the ground surface) using a screw auger of 31 mm diameter. We changed the initial location of three tubes, because we could not core due a very hard substrate—probably large belowground roots. All of the tubes were installed vertically in the soil (90°).

### Comparing EnRoot with commercial minirhizotrons

EnRoot was compared with 3 other commercial minirhizotrons in terms of nine characteristics that we deemed to be important, such as camera resolution, weight and price (see Table 2 for full list). Details of the commercially-available minirhizotrons were provided by suppliers, manufacturers’ online documentation, and peer-reviewed publications.

## Supplementary information

**Additional file 1.** Bash script to correct image distortion with a how-to-use guide and Fig. S2.1.

**Additional file 2.** 3D files for printing the components of EnRoot with a quick guide.

**Additional file 3.** Video of mangrove roots development recorded with EnRoot imaging device.

## Data Availability

The material and dataset supporting the conclusions of this article are included within the article and its additional files.
