# Supplementary material for: EnRoot: a narrow-diameter, inexpensive and partially 3D-printable minirhizotron for imaging fine root production
Source: Plant Methods. 2019 Aug 28;15:101. doi: 10.1186/s13007-019-0489-6 (PMC6712814; doi:10.1186/s13007-019-0489-6)
Supplement: Supplementary file 1 — Additional file 1. Bash script to correct image distortion with a how-to-use guide and Fig. S2.1. [file 13007_2019_489_MOESM1_ESM.zip › AdditionalFile_1/AF_1_Guide_bash_script.docx]

**Supplementary material 1: Running EnRoot’s bash script on Windows 10**

The bash script can be found in the ESM_2 and in github: <https://github.com/jonnyhuck/EnRoot_bashscript>

Please note: The bash script has been developed for EnRoot’s design. However, the points file can be tailored using QGIS.

1. Install **Ubuntu** on Windows 10 as a **windows subsystem for Linux**. The instructions are here <https://docs.microsoft.com/en-us/windows/wsl/install-win10>

See also <https://docs.microsoft.com/en-us/windows/wsl/user-support> for information on setting up a Ubuntu **user account and permissions**.

| 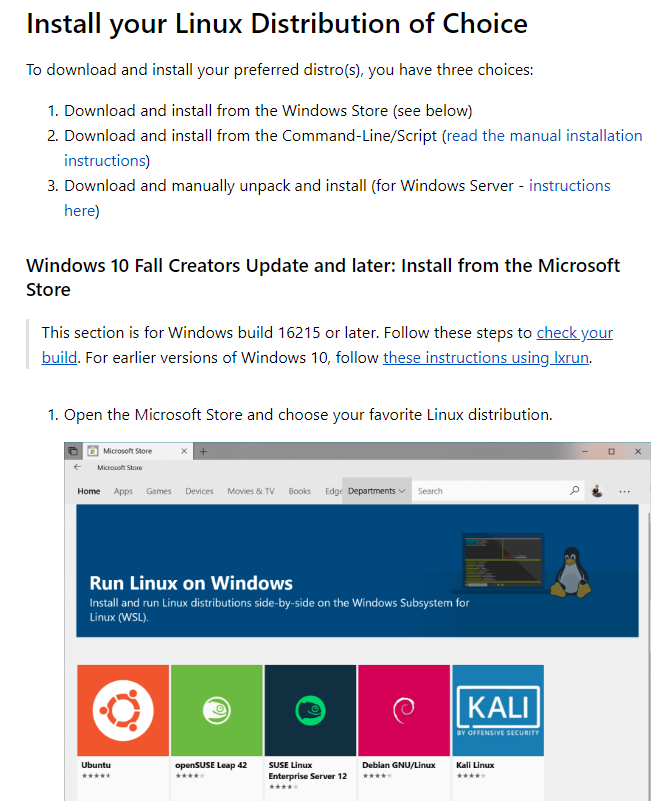 |
| --- |

1. Install **GDAL** via Ubuntu. The instructions can be found here <http://www.sarasafavi.com/installing-gdalogr-on-ubuntu.html>
2. It should be possible to open Ubuntu from the Start menu in Windows 10 or by typing **wsl** in the Windows command prompt (or Windows PowerShell). The Windows shell can be used to control Ubuntu.


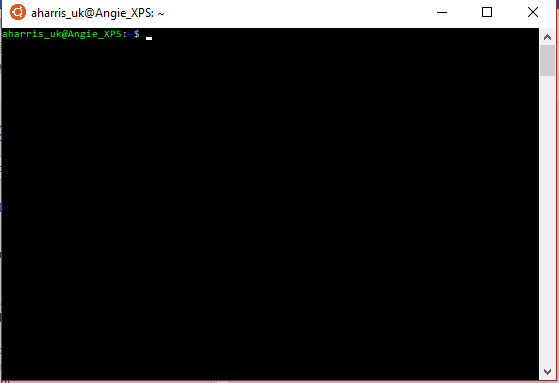


1. The following pages may be helpful for those not familiar with Linux commands

- How do I access my C drive? <https://docs.microsoft.com/en-us/windows/wsl/faq>
- Moving between directories etc
   <https://help.ubuntu.com/community/UsingTheTerminal>

1. Create a folder and place the EnRoot bash script and the images to be corrected **in the same folder** as each other. To make things as easy as possible, avoid long file pathnames (i.e., avoid putting the folder deep within the Windows system) and avoid using spaces when naming a folder. For example, a folder named ‘my fieldwork photos’ should be named my_fieldwork_photos with no spaces.
2. In the bash shell, navigate to the directory containing the new folder. Consider the example where the data have been placed in a Windows directory **C:\Users\Angela\Documents**. To get there in Ubuntu, type the address as

**cd /mnt/c/Users/Angela/Documents**

1. Type **pwd** to see the file path or **ls** to see the list of files to check the directory is the correct one.
2. Now make the **warp.sh** script “executable” (i.e. enable it to run) by typing

**chmod +x ./warp.sh**

1. Now the script can be run (again assuming that the user has correctly navigated to the directory where the files are located, as outlined in the steps above) using

**./warp.sh**

The script can be run each time new images are added to the directory (it is not necessary to install Ubuntu and GDAL again, or to make the script executable again).

The script can also be run directly from Windows without opening the Ubuntu shell. To do that, follow the instructions on this website <https://www.howtogeek.com/265900/everything-you-can-do-with-windows-10s-new-bash-shell/>.

10. Results of the geometric transformation of EnRoot pictures (Fig. ESM2_1)


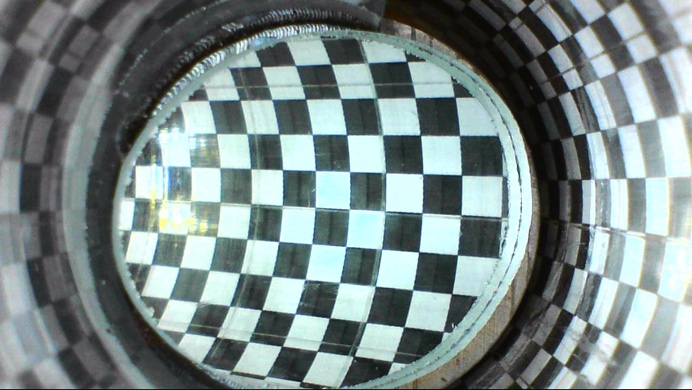

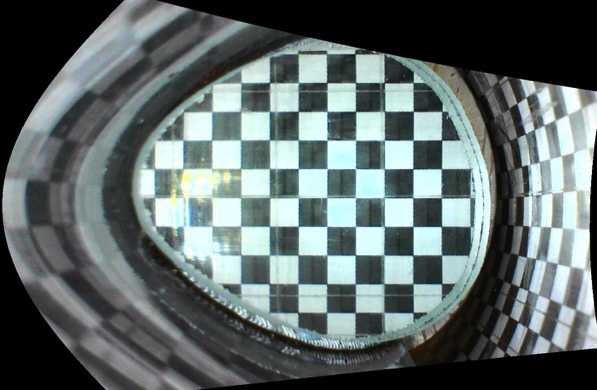


Fig. AF1_1: An illustration of a captured image from a calibration grid before (left) and after (right) transformation using the EnRoot’s bash script.
