# Supplementary figures and images for: EnRoot: a narrow-diameter, inexpensive and partially 3D-printable minirhizotron for imaging fine root production
Source: Plant Methods. 2019 Aug 28;15:101. doi: 10.1186/s13007-019-0489-6 (PMC6712814; doi:10.1186/s13007-019-0489-6)

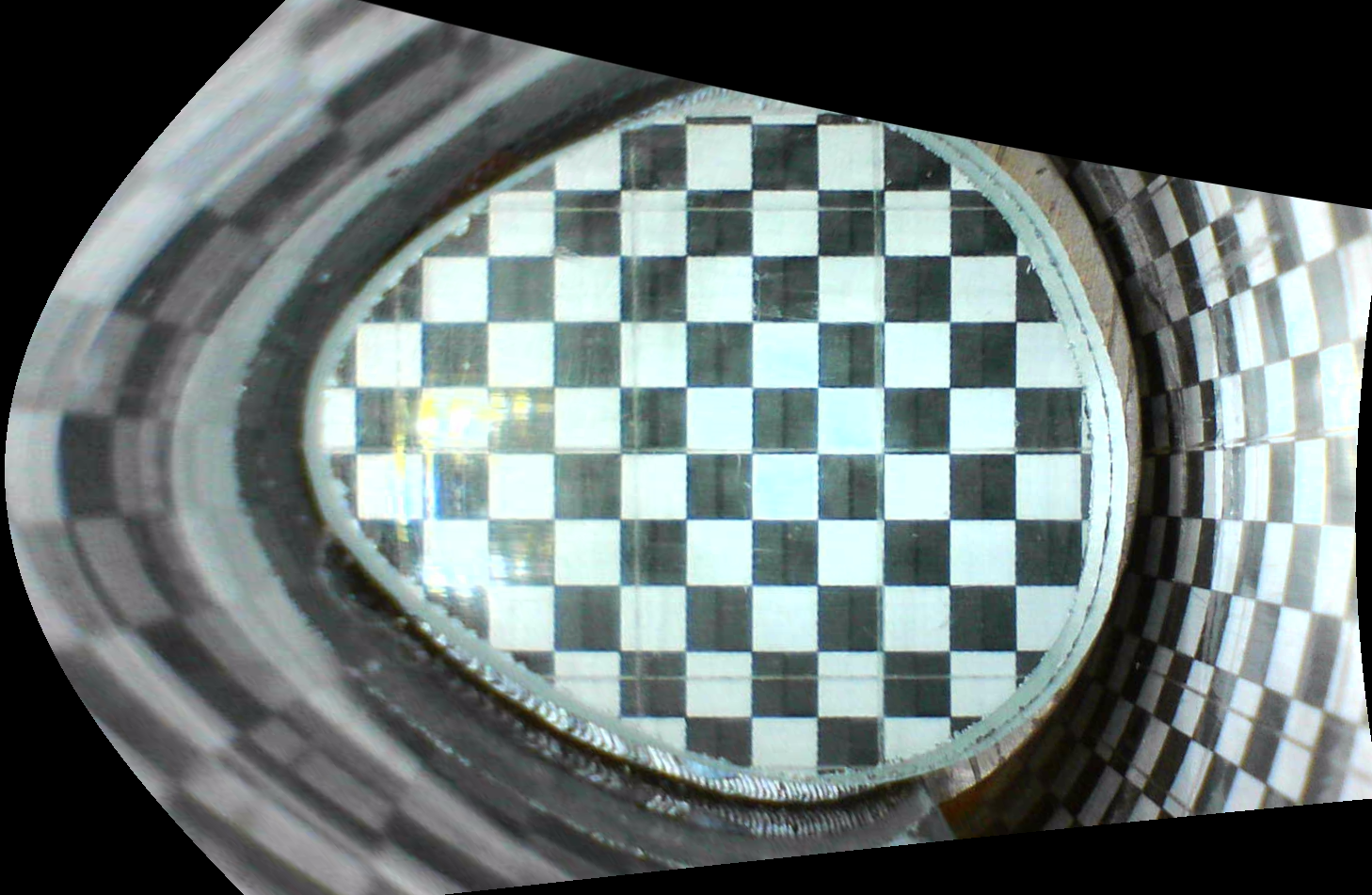

Supplement: Supplementary file 1 — Additional file 1. Bash script to correct image distortion with a how-to-use guide and Fig. S2.1. [file 13007_2019_489_MOESM1_ESM.zip › AdditionalFile_1/AF_1_EnRoot_bash_script/out/Snap_0001.tif]

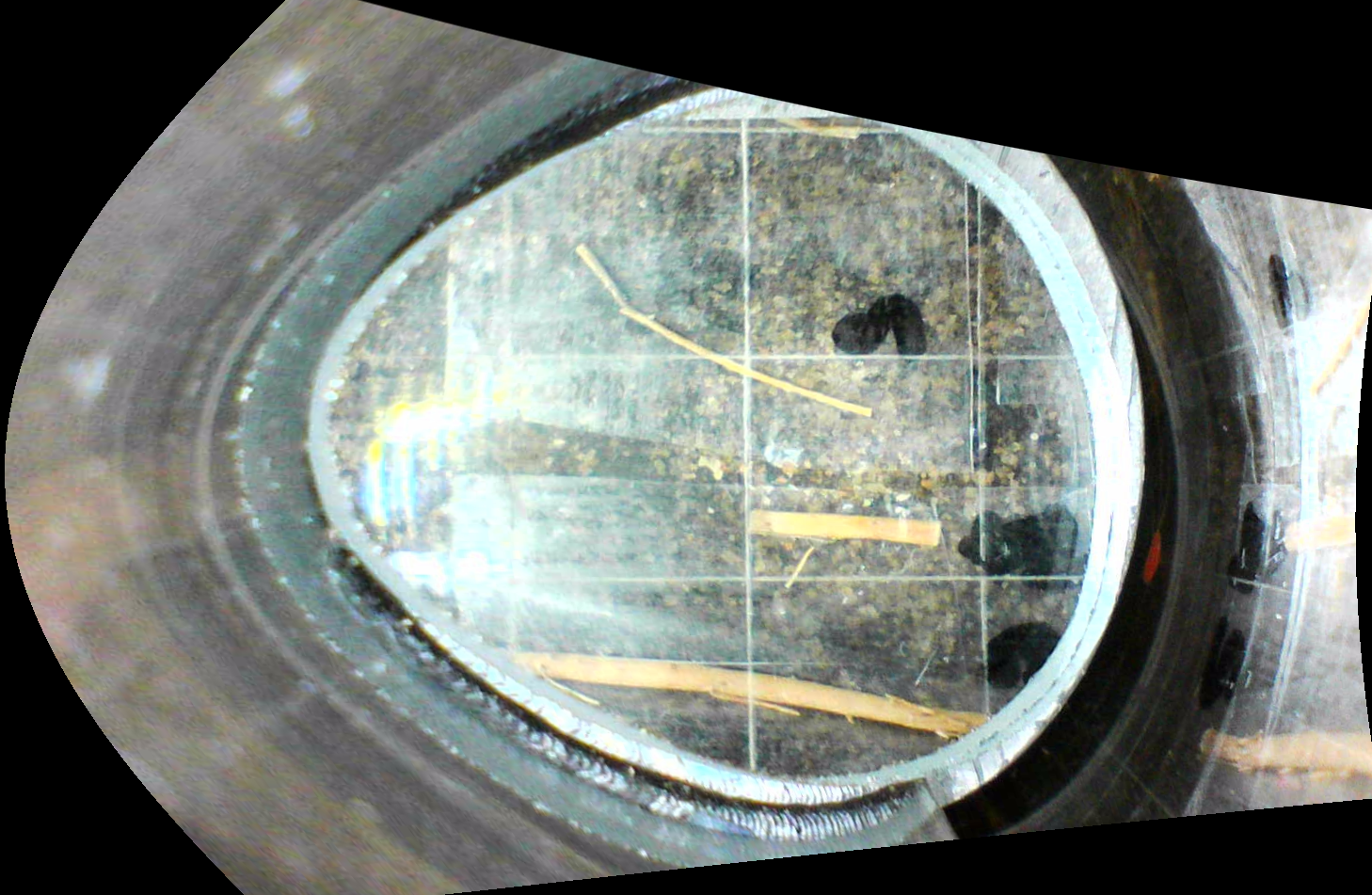

Supplement: Supplementary file 1 — Additional file 1. Bash script to correct image distortion with a how-to-use guide and Fig. S2.1. [file 13007_2019_489_MOESM1_ESM.zip › AdditionalFile_1/AF_1_EnRoot_bash_script/out/Snap_002.tif]

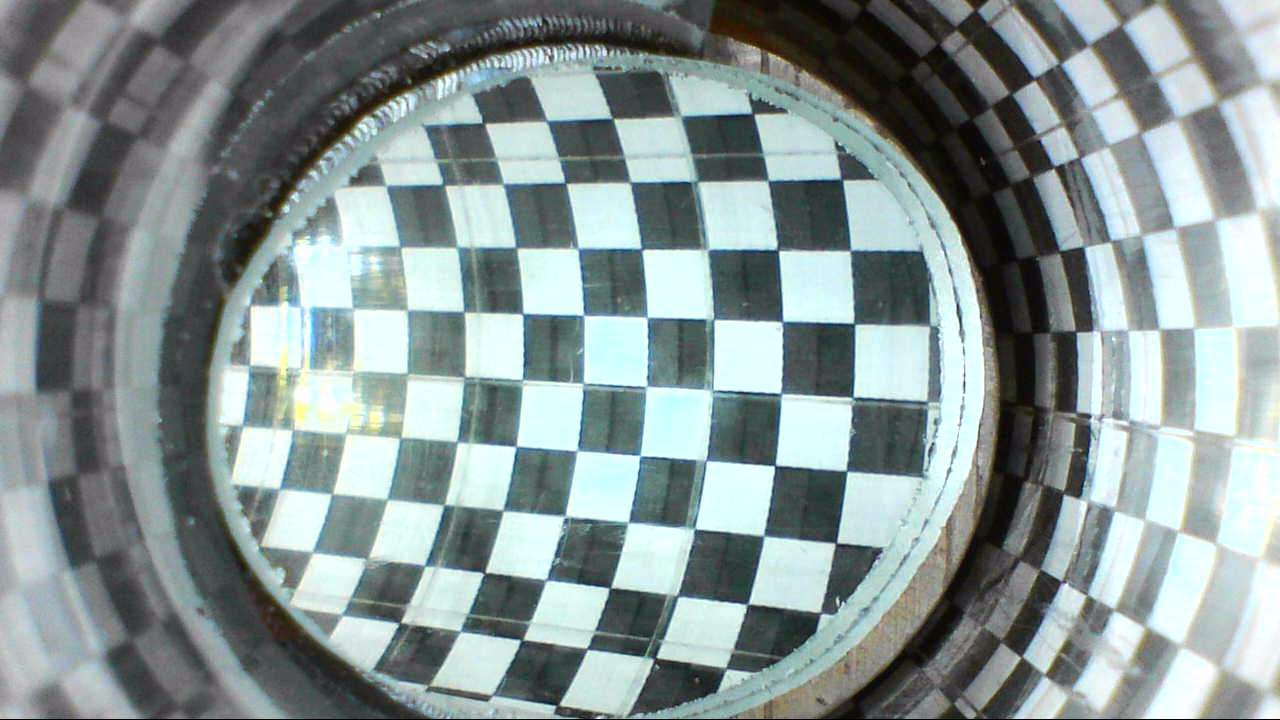

Supplement: Supplementary file 1 — Additional file 1. Bash script to correct image distortion with a how-to-use guide and Fig. S2.1. [file 13007_2019_489_MOESM1_ESM.zip › AdditionalFile_1/AF_1_EnRoot_bash_script/Snap_0001.jpg]

**Supplementary material 2: quick 3D printing guide**

1


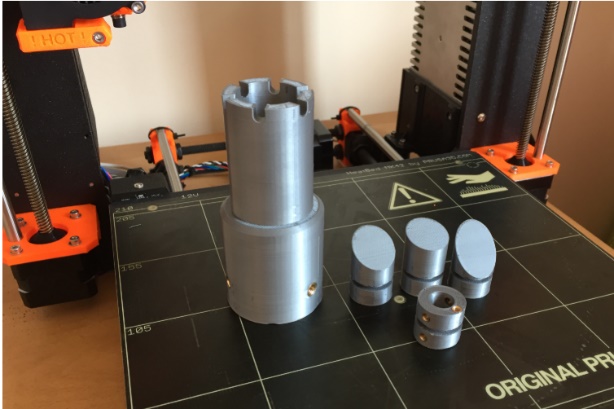

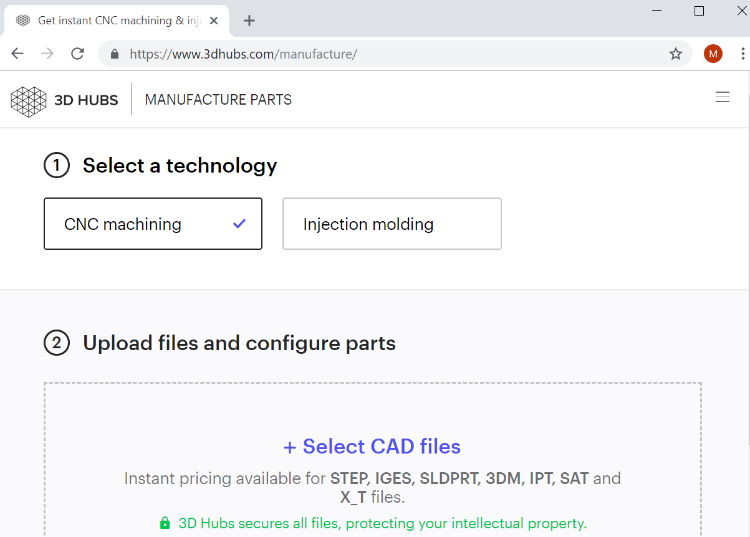


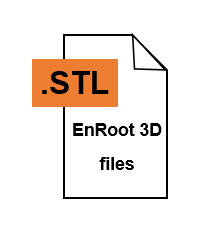


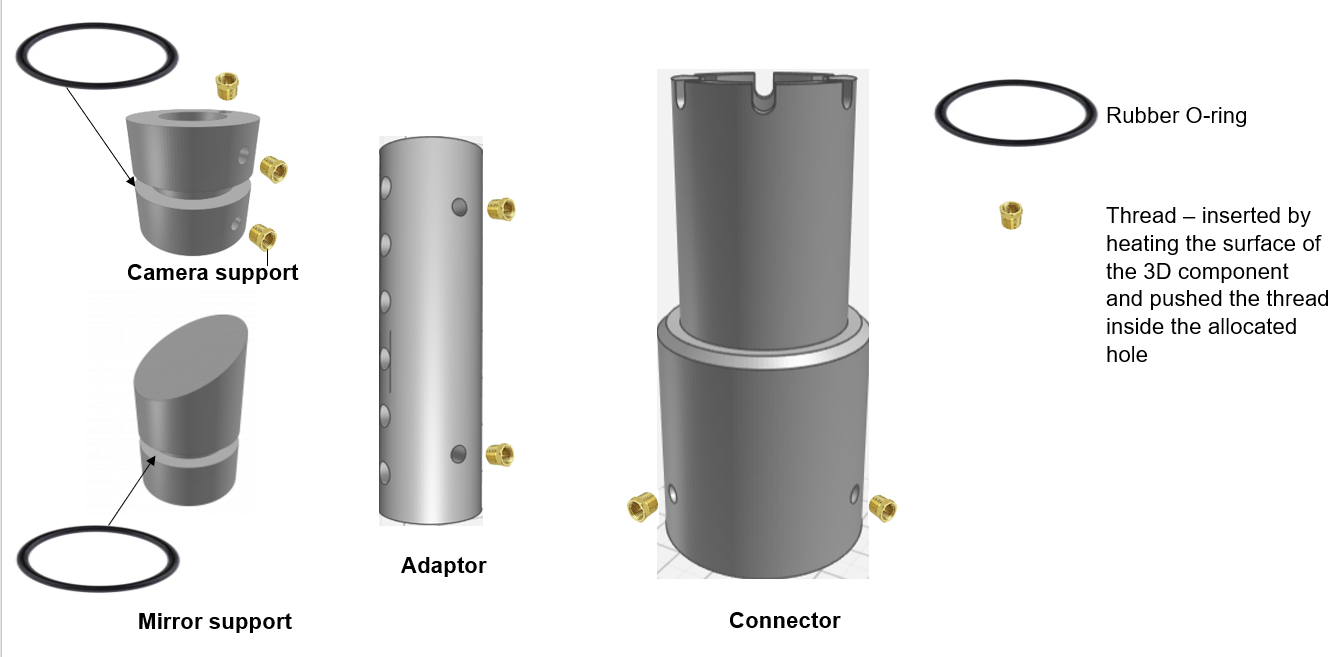


Build Enroot components

3D print files

2

Supplement: Supplementary file 2 — Additional file 2. 3D files for printing the components of EnRoot with a quick guide. [file 13007_2019_489_MOESM2_ESM.zip › AdditionalFile_2/AdditionalFile_2_quick 3D printing guide.docx]
